# 591. Spotlight Effect: A Nurse-led Engagement Tool to Strengthen the Burn Interprofessional Team

**DOI:** 10.1093/jbcr/irag033.385

**Published:** 2026-04-06

**Authors:** Stacey Richerbach, Karen J Richey, Kevin N Foster

**Affiliations:** Diane & Bruce Halle Arizona Burn Center - Valleywise Health, Phoenix, AZ; Diane & Bruce Halle Arizona Burn Center - Valleywise Health, Phoenix, AZ; Diane & Bruce Halle Arizona Burn Center - Valleywise Health, Phoenix, AZ

## Abstract

**Introduction:**

Specialty burn units rely on cohesive teams to deliver optimal care. While clinical skills are critical, belonging, mutual respect, and recognition strongly influence morale and function. The importance of team cohesion and role clarity within our Center became clear following staff turnover and the onboarding of new and contract personnel. In response, nurse leaders implemented an Employee Spotlight (ES) to enhance approachability, strengthen connections, and promote role clarity. This quality improvement project evaluated staff perception of ES impact on connection, approachability, and cohesion.

**Methods:**

Over two years, Burn RNs, techs, therapists, APPs, Attendings, and support staff, were invited to participate in the ES. For each featured individual, an email was disseminated to roughly 200 Burn employees, and an employee-specific ES poster was placed in the conference room and staff lounges. At the bottom of each ES feature email, members of the interprofessional team were invited to share their information, including role, hometown, education, and a headshot for visual recognition. Participants answered 8–10 of 35 pre-selected questions on personal favorites, hobbies, and self-reflection to foster connection. After the 27th ES, a 19-item anonymous survey with positive (POS), neutral, and negative (NEG) phrasing was distributed via Forms. Questions were categorized by approachability, connection/cohesion, recognitioN/Awareness, and overall impact. Descriptive statistics were performed.

**Results:**

Thirty team members responded. Approachability had the highest POS association (71.7%), followed by connection/cohesion (71.3%), recognitioN/Awareness (67.5%), and overall impact (58.3%). Most (86.7%) agreed that “reading about colleagues’ backgrounds makes me feel more connected to the team.” Similarly, 83.3% agreed that “learning personal details makes colleagues more relatable” and that ES “helped me recognize shared interests or experiences.” Qualitative feedback to open-ended questions reflected enjoyment in learning about colleagues, improved visual recognition, and greater awareness of team members’ respective roles.

**Conclusions:**

Nurse leaders positively impacted team dynamics through ES implementation. A structured ES program offers a low-cost, high-impact strategy to foster teamwork and engagement in specialty units. Ongoing implementation and expanded questions may further enhance satisfaction and retention while reinforcing culture.

**Applicability of Research to Practice:**

Regular recognition through ES is a scalable tool to enhance teamwork and engagement across teams. Further evaluation is needed to assess effects on staff satisfaction and long-term retention.

**Funding for the study:**

N/A.

